# Commentary: Selective Fiber Degeneration in the Peripheral Nerve of a Patient With Severe Complex Regional Pain Syndrome

**DOI:** 10.3389/fnins.2019.00019

**Published:** 2019-02-05

**Authors:** Lakshmi Vas

**Affiliations:** Ashirvad Institute for Pain Management and Research, Mumbai, India

**Keywords:** complex regional pain syndrome, ultrasound-guided dry needling, neuromyopathy, myofacial pain, myofacial trigger points, reflex sympathetic dystrophy

Complex regional pain syndrome (CRPS), a chronic pain condition affecting the extremities, can severely affect the quality of life. Beyond a consensus that CRPS arises from damage to the central and peripheral nervous systems, its etiopathology is poorly understood. In a recent publication in *Frontiers in Neuroscience*, Yvon et al. (2018) examined radial, median, and ulnar nerve specimens from a CRPS-affected amputated limb and showed widespread (47–58%) selective degeneration in the larger myelinated Aα fibers (motor/proprioception) and in groups of small unmyelinated C fibers (Remak bundles), while the smaller Aδ (pain/temperature) fibers were spared.

The authors postulated that large Aα fiber degeneration could consequently affect the associated innervated muscles. These findings are of interest to us as they provide a mechanistic explanation for the profound structural disruption we have observed in the muscles of CRPS-affected limbs using musculoskeletal ultrasound (MSKUSG) (Vas and Pai, 2012, 2014, 2016b; Vas et al., 2013, 2016c, 2018; Pai and Vas, 2018). The sonographic signature of normal muscle is fairly distinct with a well-defined hyperechoic epimysium with an echolucent (dark) background with bright punctate and curvilinear echoes of the perimysium that appear as bright streaks [Figure 1A, left panels; (Walker et al., 2004)]. In contrast, MSKUSG of the CRPS muscle reliably shows an extreme increase in echogenicity (“snowstorm-like” appearance) that is indicative of muscle fibrosis (Reimers et al., 1993), with loss of muscle outlines [Figure 1A, right panels; (Vas et al., 2013)]. In severe cases, several muscles coalesce together to form one indistinct mass of hyperechogenecity (Figure 1A, left panels), with a marked loss of muscle bulk compared with the normal limb, as indicated by caliper measurements (Vas et al., 2016c). Histopathological changes in the muscle in CRPS have been previously reported (van der Laan et al., 1998; Hulsman et al., 2009), and other studies have shown a robust correlation between histological and sonographic changes in muscle in patients with inflammatory myopathy (Reimers et al., 1993; Pillen et al., 2009), suggesting that the observed sonographic changes in the muscles of CRPS patients (Vas et al., 2013, 2016c) may be accompanied by histological changes.

**Figure 1:**
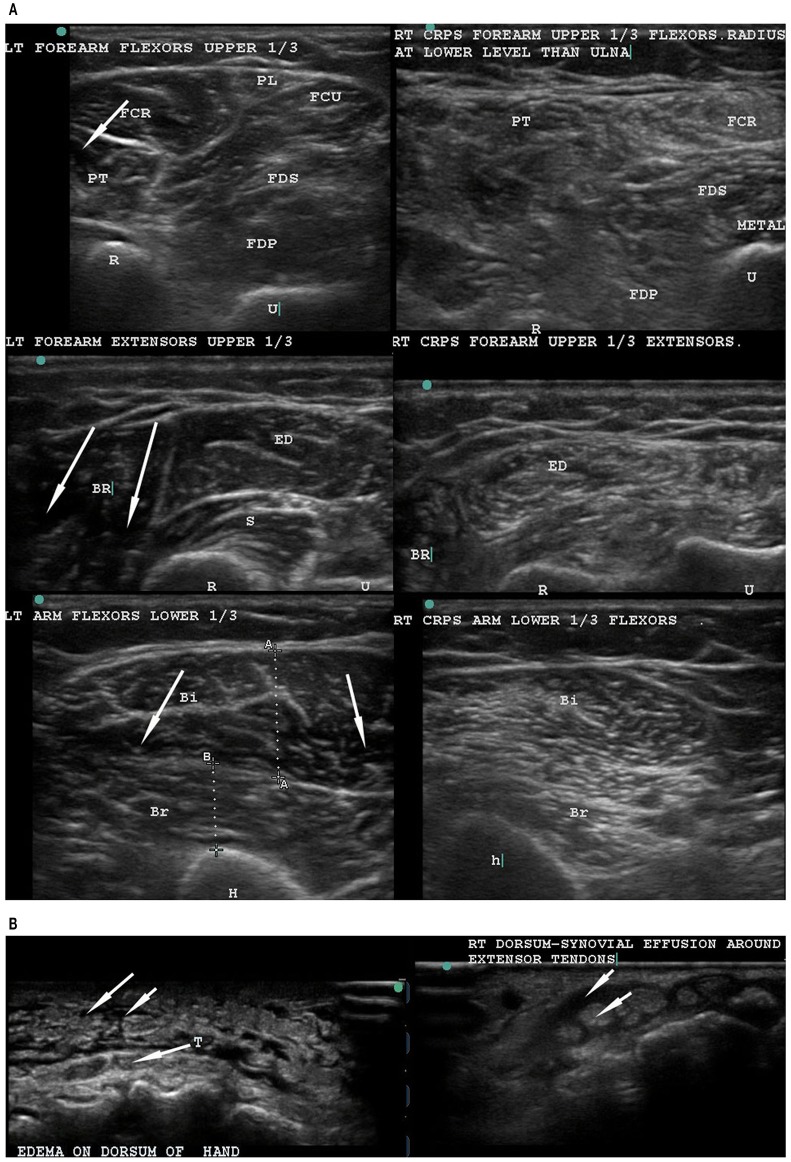
Musculoskeletal ultrasonography to compare the normal left arm with the CRPS-affected right arm. **(A)** Musculoskeletal ultrasound of the normal left forearm (left) and the CRPS-affected right forearm (right) of a single patient. The normal left extremity muscles have well-defined outlines, and the muscle is hypoechoic (dark spaces, see arrows) with bright curvilinear echoes. In contrast, the CRPS-affected right arm and forearm muscles show general hyperechogenecity (as indicated by a snowstorm-like appearance not seen in normal muscle), with loss of the dark spaces as well as muscle outlines. **(B)** Ultrasonography of the dorsum of hand in a non-CRPS patient (left) and a CRPS patient (right). In the non-CRPS patient, the edema is subcutaneous (left, upper arrows) and superficial to the tendons (T) (arrow from T). In the CRPS-affected hand, a peritendinous tenosynovial effusion (right, upper arrow) surrounds the tendon (right, lower arrow). Note the normal narrow subcutaneous space without any fluid accumulation. T, tendon.

Current treatments for CRPS such as neuromodulator pharmacotherapy and sympathetic ganglion block exclusively address nerve pathology, with limited results (Day, 2008; Gierthmühlen et al., 2014; Baig et al., 2017; Stanton-Hicks, 2018; Urits et al., 2018; Zyluk and Puchalski, 2018). Our approach has instead addressed muscle dysfunction through dry needling, a treatment where needles of 32-G are placed at 1 cm intervals along both the length and breadth of muscles. Dry needling has been shown to deactivate MTrPs and relieve pain in a variety of conditions (Tsai et al., 2010; Mayoral et al., 2013; Rainey, 2013; Mejuto-Vázquez et al., 2014; Rock and Rainey, 2014; Salom-Moreno et al., 2015; Shah et al., 2015). We have developed a more comprehensive and extensive dry needling protocol (discussed later), carried out under ultrasound guidance. As previously reported by our group (Vas and Pai, 2012, 2014, 2016a; Pai and Vas, 2018; Vas et al., 2018), this protocol of ultrasound-guided dry needling (USGDN) has shown remarkable success, producing not only sustained pain relief, but also disability relief, as indicated by improvement in the Disability of Arm, Hand, and Shoulder scale (Vas and Pai, 2016a,b). Importantly, USGDN-treated patients are able to routinely reassume their previous lifestyles and professions (Vas and Pai, 2012, 2014, 2016a; Vas et al., 2018).

We have also shown that this muscle fibrosis observed very consistently using MSKUSG in CRPS is not seen in other neuropathic pain conditions (Vas and Pai, 2016b). Strikingly, following CRPS reversal with USGDN, the profound structural disruption observable by MSKUSG is also reversed, with a return to normal muscle appearance (Vas et al., 2016c).

Based on findings of muscle changes and the routine reversal of muscle changes as well as CRPS symptoms, we propose that the primary pathology of CRPS is in the muscle, in the form of a co-contraction of the agonist and antagonist muscles responsible for performing the digital, wrist, elbow, and shoulder movements. We hypothesize that the abnormal co-activation of digital flexor-extensor and pronator-supinator muscles replaces the normal inbuilt reciprocal inhibition of agonist and antagonist muscles. Attempted movements of abnormally co-contracted muscles causes friction between the tendons and the tenosynovial sheaths, leading to the clinical presentation of tenosynovitis of all digital tendons. We have come to conclude that it is this very much exaggerated deQuervain-like involvement of all the flexor and extensor tendons that gives rise to all the manifestations of CRPS, including the sensory, motor, sudomotor, and vasomotor symptoms and signs (Reimers et al., 1993; van der Laan et al., 1998; Hulsman et al., 2009; Pillen et al., 2009; Vas and Pai, 2016b; Vas et al., 2016a,c). In fact, CRPS was mistakenly treated as deQuervain's tenosynovitis in a patient who later recovered completely after USGDN (Vas and Pai, 2016a). We have also found that MSKUSG clearly demonstrates the difference between tenosynovial effusion in the swollen CRPS hand (Figure 1B, left) from simple subcutaneous edema in a non-CRPS patient (Figure 1B, right).

USGDN is a simple yet effective technique that resolves the CRPS symptoms in a step-by-step manner. It would be easy for physicians to acquire the requisite skill by a thorough re-grounding in anatomy, sonoanatomy and the technique of needle placement in muscles under ultrasound guidance. However, USGDN reported in our publications should not be equated with the dry needling presently practiced in the US, Europe and elsewhere (Janz and Adams, 2011; Liu et al., 2015; Gattie et al., 2017; Tejera-Falcón et al., 2017; Rastegar et al., 2018). There are many important differences, including the number of needles (6 vs. 30–60 needles), length of needles (25–50 vs. 13–120 mm), duration of needle maintenance in muscle (<1 vs. 30 min), maximum sessions (6 vs. 20), and method (blind unguided vs. ultrasound-guided). We believe that these differences, particularly ultrasound guidance, the number of needles, and duration of maintenance of needles in muscles, may contribute to the effectiveness of USGDN at our clinic in treating myriad conditions, including CRPS (Vas and Pai, 2012, 2014, 2016a; Vas et al., 2014a,b, 2015, 2016a,b,c, 2018).

In summary, we believe that the neural changes observed by Yvon et al. (2018) may be responsible for the muscle changes, but it is these muscle changes that form the primary pathology of CRPS. Indeed, degeneration of the larger Aα (motor/proprioception) fibers might be instrumental in producing the constant unrelenting muscle co-contraction that leads to generation of MTrPs. To our knowledge, no other groups have used MSKUSG to study CRPS, nor used USGDN as a specific treatment for CRPS. Exploration of these observations by other groups is necessary. Lastly, given the evidence implicating muscle pathology in CRPS, the suitability of USGDN (a remarkably safe technique) as a first-line option should be further explored, especially before considering more controversial approaches such as limb amputation.

## Author Contributions

The author confirms being the sole contributor of this work and has approved it for publication.

### Conflict of Interest Statement

The author declares that the research was conducted in the absence of any commercial or financial relationships that could be construed as a potential conflict of interest.
